# ANKS1B Interacts with the Cerebral Cavernous Malformation Protein-1 and Controls Endothelial Permeability but Not Sprouting Angiogenesis

**DOI:** 10.1371/journal.pone.0145304

**Published:** 2015-12-23

**Authors:** Stefanie E. Herberich, Ralph Klose, Iris Moll, Wan-Jen Yang, Joycelyn Wüstehube-Lausch, Andreas Fischer

**Affiliations:** 1 Vascular Signaling and Cancer (A270), German Cancer Research Center (DKFZ-ZMBH Alliance), D-69120, Heidelberg, Germany; 2 Vascular Biology and Tumor Angiogenesis, Medical Faculty Mannheim (CBTM), Heidelberg University, D-68167, Mannheim, Germany; 3 Department of Medicine I and Clinical Chemistry, Heidelberg University Hospital, D-69120, Heidelberg, Germany; BloodCenter of Wisconsin, UNITED STATES

## Abstract

Cerebral cavernous malformations are fragile blood vessel conglomerates in the central nervous system that are caused by mutations in the *CCM1/KRIT1*, *CCM2* or *CCM3* genes. The gene products form a protein complex at adherens junctions and loss of either CCM protein disrupts endothelial cell quiescence leading to increased permeability and excessive angiogenesis. We performed a yeast 2-hybrid screen to identify novel proteins directly interacting with KRIT1. The ankyrin repeat and sterile alpha motif domain-containing protein 1B (ANKS1B) was identified as a novel binding partner of KRIT1. Silencing of ANKS1B or the related gene ANKS1A in primary human endothelial cells had no significant effects on cellular proliferation, migration and sprouting angiogenesis. However, silencing of ANKS1B expression disturbed endothelial cell barrier functions leading to increased permeability. Forced ANKS1B expression reduced permeability. This was independent of Rho kinase activity and the presence of KRIT1. Taken together, ANKS1B was identified as a novel KRIT1-interacting protein that selectively controls endothelial permeability but not angiogenesis.

## Introduction

Cerebral cavernous malformations (CCMs) affect approximately 1 in 250 humans. CCMs can lead to a variety of clinical manifestations, most commonly headaches, seizures, and stroke, depending on size, growth rate and localisation [1]. The inherited form of the disease is caused by loss-of-function mutations in the *KRIT1* (CCM1), *CCM2* (Malcavernin) or *CCM3* (PDCD10) genes [2,3]. *CCM* mutations primarily affect endothelial cells causing decreased endothelial junctional stability and excessive angiogenesis [4–11].

CCM proteins have been detected at the membrane, in the nucleus and associated to microtubules. Most importantly a cluster of all three proteins is enriched at adherens junctions of endothelial cells [2,3]. This is essential to restrict vascular permeability via control of Rap1, RhoA and Rho kinase signalling [9,10,12–16]. Secondly, KRIT1 interacts with ICAP1 (also known as ITG1BP1), to control endothelial cell adhesion to the extracellular matrix [17] and the beta1-integrin activation status [18]. KRIT1 is also needed to restrict endothelial-to-mesenchymal transition and Notch signalling [11,19]. Both processes are integral to prevent uncontrolled angiogenesis.

CCM proteins do not possess enzymatic activity and it is believed that they act via protein-protein interactions [20]. KRIT1 recruits the phosphotyrosine binding domain (PTB) of CCM2 which can bind the CCM3 protein [20–22]. Here we report the identification of the ankyrin repeat and sterile alpha motif domain-containing protein 1B (ANKS1B) as a novel KRIT1 binding protein.

ANKS1B (also known as EB-1, AIDA-1, cajalin-2, ANKS2) contains one PTB domain and two sterile alpha motif (SAM) domains. It is widely expressed in brain and acts as a scaffold protein at postsynaptic densities where it plays a role for long-term potentiation (LTP), a basic mechanism for learning and memory [23]. ANKS1B interacts with the amyloid-beta protein [24], the tyrosine kinase receptor EphA8 [25], and regulates the degradation of EphA receptors [26]. The fusion protein E2a-PBX1 markedly increases its expression in pre-B cell acute lymphoblastic leukemia [27]. The related protein ANKS1A (also known as ANKS1, Odin) interacts with 14-3-3 proteins [24], restricts proliferation of fibroblasts [28]. However, its deletion in mice did not reveal any obvious developmental defects [25].

This study aimed at elucidating the functions of the newly identified KRIT1-binding protein ANKS1B in endothelial cells. Since KRIT1 is integral to inhibit angiogenesis and endothelial permeability, these two processes were studied in detail.

## Materials and Methods

### Cell culture and RNAi

Human umbilical vein endothelial cells (HUVEC) were freshly isolated from umbilical cords according to the declaration of Helsinki and with approval of the Heidelberg University ethics review board. Written informed consent from the donor was obtained. HUVEC were cultured in ECGM with supplements and 2.5% FCS (PAN Biotech, Aidenbach, Germany). For siRNA transfection 200 nmol/l annealed siRNA duplexes were mixed with 6 μl of Oligofectamine (Invitrogen, Carlsbad, CA, USA) and added to 1.2 x 10^5^ HUVEC plated in a 6-well plate. Control siRNA and two established siRNAs against ANKS1B (s32343, s32344 referred to as si-ANKS1B #1 and #2) were from Ambion (Thermo Fisher Scientific, Waltham, MA, USA). Short hairpin RNAs (SMARTpool) against ANKS1A (L-025085-01 referred as si-ANKS1A sp), KRIT1 (L-003825-00-0005 referred as si-KRIT1) and ROCK1 (L-003536-00-0005 referred as si-ROCK1) were from Dharmacon, GE Healthcare, UK.

### Endothelial cell migration, proliferation and sprouting

A HUVEC monolayer was scratched with a pipette tip 48 h after siRNA transfection and closure of the wounded area was monitored (Olympus IX inverted microscope with Cell^P software). To measure cell proliferation, HUVEC were labelled with 5-bromodeoxyuridine (BrdU) and 24 h later the incorporation of BrdU in the newly synthesized DNA was determined (Cell Proliferation ELISA, Roche, Mannheim, Germany). The spheroid-based sprouting assay in collagen was performed as described [13].

### Yeast 2-hybrid screening and LUMIER assay

Full-length human KRIT1 cDNA [11] was subcloned into pGBKT7 and used as bait for screening cDNA libraries derived from human adult and fetal brain (Clontech, Takara Bio, Mountain View, CA, USA). Protein interaction *in vivo* was determined by LUminescence-based Mammalian IntERactome mapping (LUMIER) [26]. Full length human KRIT1 [11] and ANKS1B (Genbank No. EL736896) cDNA was inserted into pcDNA3-Rluc-GW (fusion with N-terminal luciferase) and pTREX-dest30-ntPrA (fusion to N-terminal Protein A) by Gateway cloning (Invitrogen, Carlsbad, CA, USA). HEK293 cells were transiently transfected with Protein A-tagged ANKS1B and KRIT1 fused to Renilla luciferase. Cells were lysed two days after transfection and Protein A-tagged proteins purified with immunoglobulin-coated magnetic beads. Luciferase-tagged protein was detected by luminescence activity.

### Quantitative Real-Time-PCR

Total RNA was isolated with the RNeasy Kit (Qiagen, Hilden, Germany) and transcribed into cDNA (High Capacity cDNA Reverse Transcription Kit; Applied Biosystems, Foster City, CA, USA). cDNA was mixed with POWER SYBR Green Master Mix and qPCR performed using an ABI StepOnePlus cycler (Applied Biosystems, Foster City, CA, USA). The housekeeping genes HPRT1, SRP14 and OAZ1 were used for normalization. Primers for realtime analysis are listed in S1 Table. All experiments included two technical and three biological replicates.

### Transendothelial electrical resistance

Transendothelial electrical resistance and corresponding capacity (ccl) were measured with a CellZscope apparatus (nanoAnalytics, Münster, Germany). 2.4 x 10^4^ HUVEC were transfected in six-well plates. The day after transfection, cells were trypsinised and plated at confluent density on fibronectin-coated transwells (3 μm pore size). After 24 hours, the medium was changed and cells were incubated at 37°C for 10 minutes before starting to measure TER for 40 hours.

### RhoA/Rac1 activation assays

HUVEC protein lysates were harvested 72 hours after siRNA transfection. Levels of activated RhoA and Rac1 were determined using the RhoA (Cat. #BK124) and Rac1 (Cat. #BK128) G-LISA^™^ Activation Assay kit (Cytoskeleton Inc., Denver, CO), according to the manufacturer's instructions. In all cases, constitutively active RhoA protein provided in the G-LISA^™^ kit, was used as a positive control to validate that the assay was functioning appropriately.

### Expression of recombinant GST-fusion proteins

Plasmids encoding for GST-NPxY-F1, -F2 and -F3 fusion proteins [29] were transformed into competent *E*.*coli* BL21 cells and cultured in LB medium (contents/L: NaCl 10 g, tryptone 10 g, yeast extraction 5 g) at 37°C until cells reached an OD_600_ of 0.8. For the expression of GST-NPxY-F1, -F2 and -F3 proteins the cells were induced with 1 mM IPTG and cultured for 3 hours at 30°C before harvesting.

### GST pull-down assay

GST pull-down experiments were performed using the Pierce GST Protein Interaction Pull-Down Kit (Pierce, Rockford, IL, USA) according the manufacturer's instructions.

### Statistical analysis

Results are expressed as mean ± Standard Deviation. Comparisons between two groups were analyzed by two-sided t test or 1-way ANOVA followed by Tukeys test when more than two groups were analyzed. P values < 0.05 were considered as statistically significant.

## Results

### ANKS1B is a novel interaction partner of KRIT1

To identify novel interacting proteins of the cerebral cavernous malformation protein-1 (KRIT1, CCM1, Figure A in S1 File) we used full-length human KRIT1 cDNA as bait for yeast 2-hybrid screening of adult and fetal brain cDNA libraries. 25 of the 136 isolated clones appeared to be typical false-positives. Of the remaining clones, 106 encoded ICAP1, two encoded CCM2, and three clones encoded ANKS1B (S2 Table). ICAP1 and CCM2 are well known interacting proteins of KRIT1 [23]. We isolated two independent ANKS1B clones and both contained the PTB domain. KRIT1 binds to the PTB domains of ICAP1 and CCM2 [23], suggesting that this is a common mode of KRIT1-mediated protein interactions.

We verified the potential KRIT1-ANKS1B protein interaction in eukaryotic cells with luminescence-based mammalian interactome mapping (LUMIER). The LUMIER assay is based on the co-purification of two tagged proteins from transiently transfected HEK293 cells and the detection of a positive interaction by luciferase activity [26]. ANKS1B could form homodimers and it also interacted strongly with KRIT1 (Figure B in S1 File).

The binding of KRIT1 to the PTB domain of known interacting proteins is mediated by one or more of the three KRIT1 NPxY domains (F1, F2 and F3). In order to investigate if NPxY-domains of KRIT1 are required for the interaction with ANKS1B we performed pull-down assays using GST-NPxY fusion-proteins (Fisher *et al*., 2015). The experiments revealed that ANKS1B specifically binds to the NPxY-F3 domain of KRIT1 (Figure C and D in S1 File). Thus, ANKS1B was identified as a novel interacting protein of KRIT1.

### ANKS1B does not affect endothelial cell proliferation, migration and sprouting

**Fig 1:**
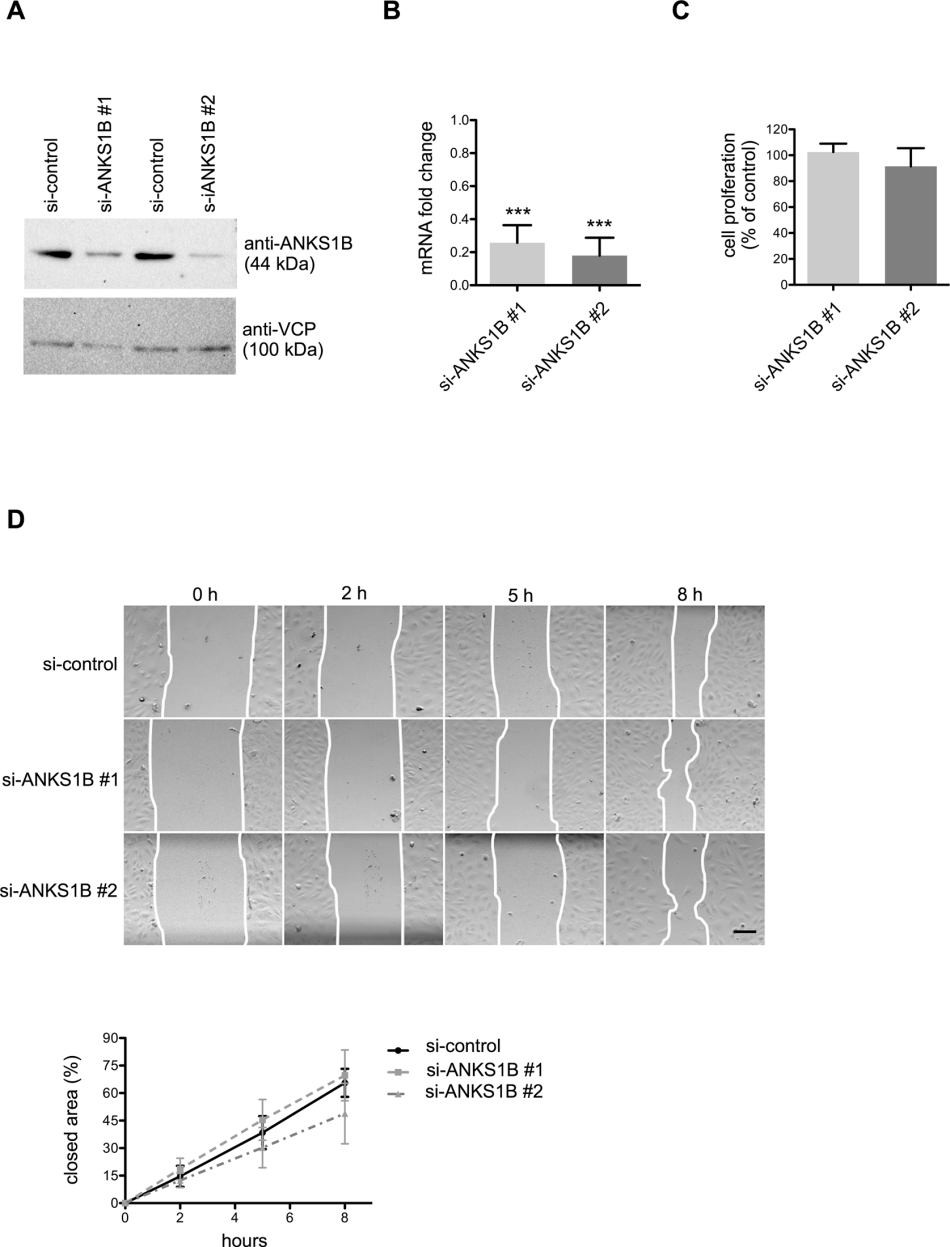
Silencing of ANKS1B does not affect endothelial proliferation and migration. (A) Representative Western blot for HUVEC protein lysates probed with anti-ANKS1B antibody after transfection with non-silencing control siRNA or two different single ANKS1B siRNAs. VCP served as protein loading control. (B) Transfection of HUVEC with two independent siRNA duplexes led to approximately 80% mRNA knockdown rates compared to non-silencing control siRNA. (C) BrdU incorporation into DNA was only slightly increased after by silencing ANKS1B expression. (D) ANKS1B knockdown did not alter the migration speed of HUVEC after wounding a monolayer. ***, p < 0.001, n = 3 experiments. Scale bar equals 100 μm. Bar graphs show mean values, error bars indicate SD.

KRIT1 is essential to keep endothelial cells in a resting, quiescent state. Silencing of KRIT1, or its interaction partner ICAP1, in primary endothelial cell of umbilical veins (HUVEC), increases migration and angiogenic sprouting [11,13]. To analyse if ANKS1B could play similar roles as KRIT1 or ICAP1, we silenced its expression in primary human endothelial cells from umbilical veins (HUVEC) with two independent siRNAs. This led to a siginificant downregulation of ANKS1B as determined by Western Blot and qRT-PCR analysis (Fig 1A and 1B).

**Fig 2:**
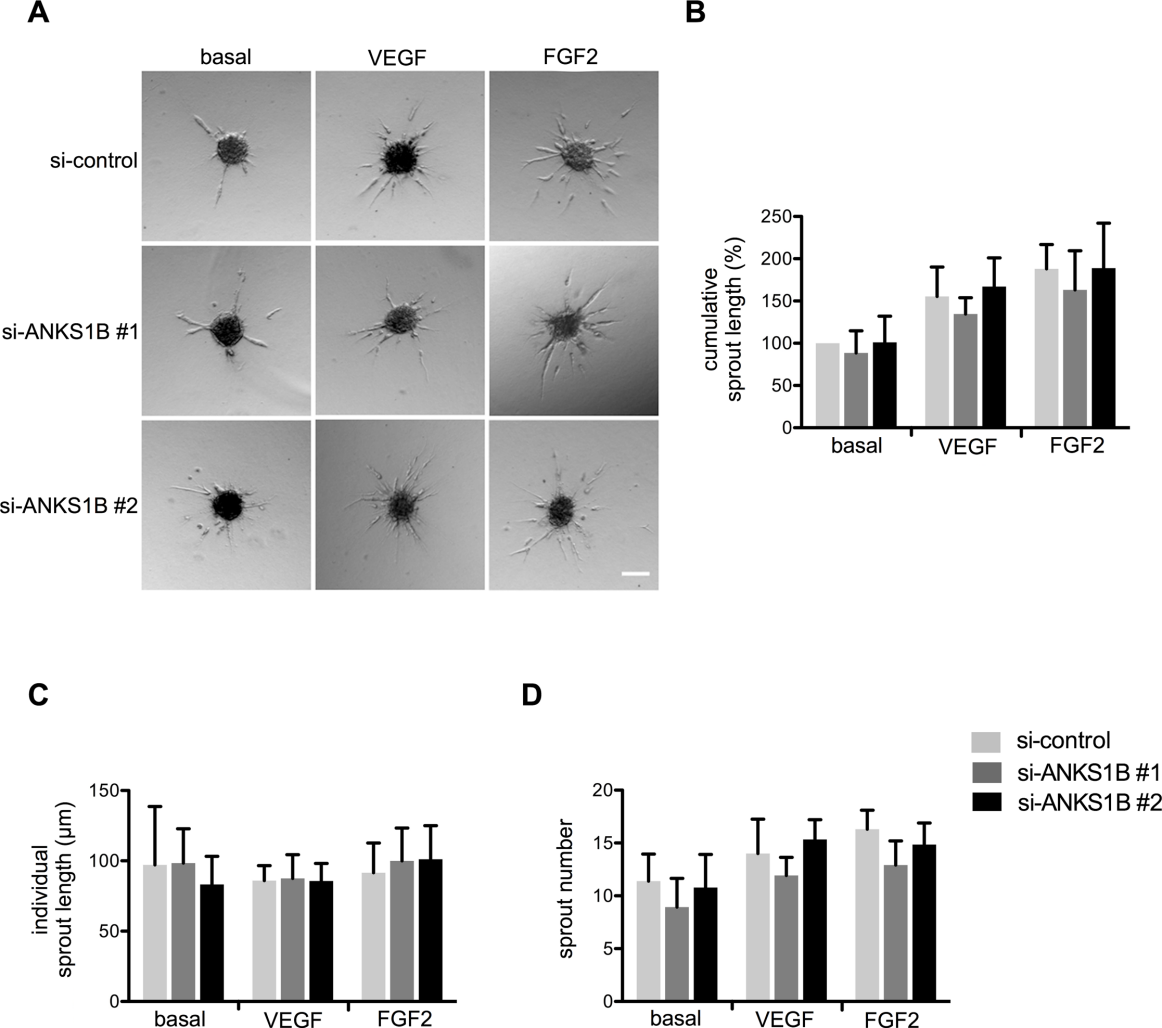
ANKS1B has no effect on endothelial sprouting. (A) HUVEC spheroids were embedded in collagen and stimulated with 25 ng/ml VEGF or FGF2. Capillary formation was measured 24 h later. Silencing of ANKS1B had no effect on cumulative sprout length (B), individual sprout length (C) and sprout number (D). N = 5 experiments. Scale bar equals 100 μm. Bar graphs show mean values, error bars indicate SD.

The proliferation rates of HUVEC were not significantly changed after silencing ANKS1B compared to control siRNA treatment as measured by BrdU incorporation (Fig 1C). Migration capacity was analysed using a scratch wound assay and silencing of ANKS1B had no significant effect on the endothelial cell migration speed (Fig 1D). Finally, the capacity of endothelial cells to form capillary-like structures when embedded as spheroids in collagen gels was analysed. Silencing of ANKS1B did not alter the number and length of sprouts under basal conditions and after VEGF or FGF2 stimulation (Fig 2A–2D). Taken together, silencing of ANKS1B had no significant effects on proliferation, migration and sprouting angiogenesis of primary human endothelial cells.

### Combined knockdown of ANKS1A and ANKS1B does not affect endothelial cell proliferation, migration and sprouting

**Fig 3:**
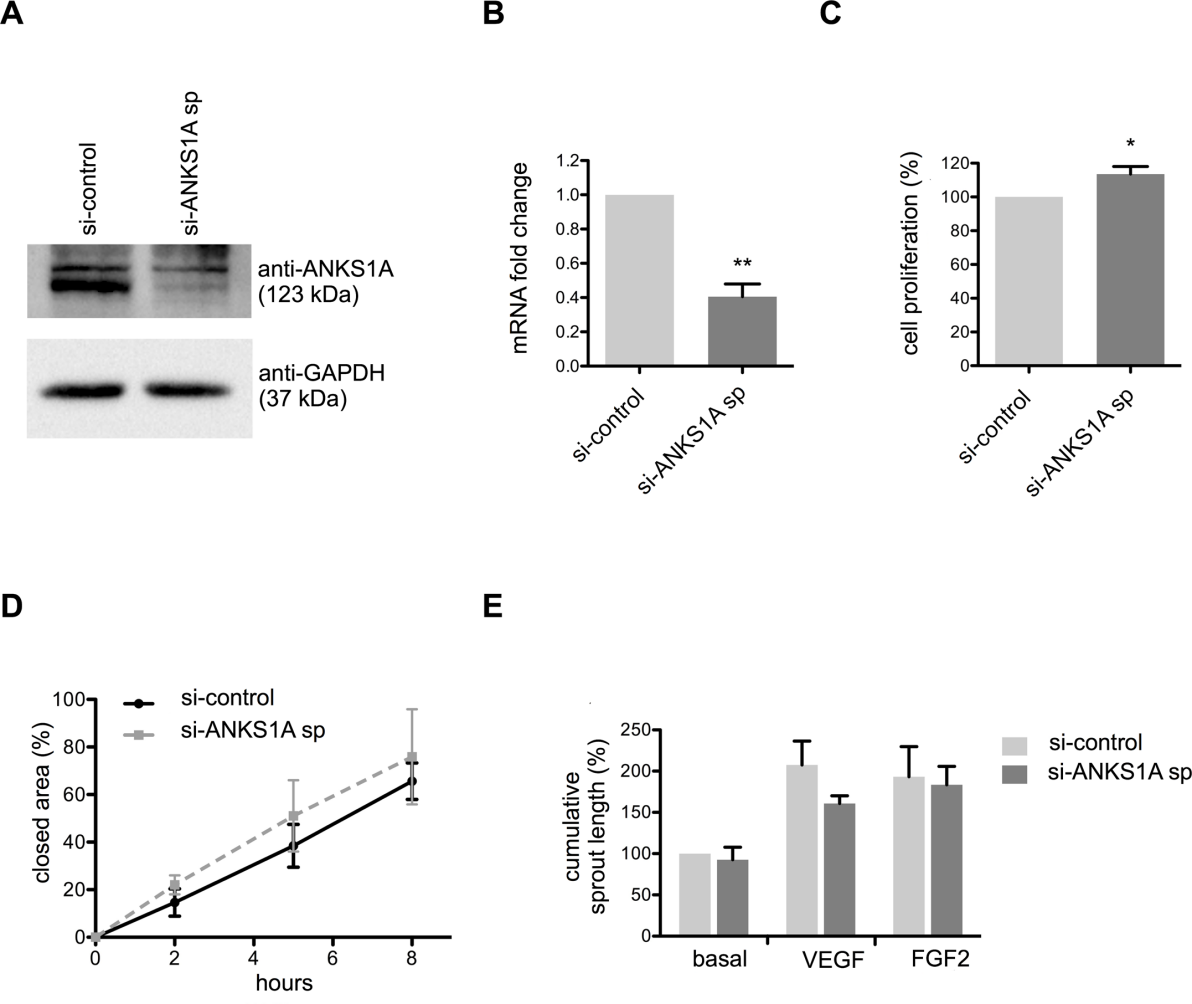
ANKS1A does not affect endothelial proliferation, migration and capillary formation. (A) Representative Western blot for HUVEC protein lysates probed with anti-ANKS1A antibody after transfection with non-silencing control siRNA or a pooled set of ANKS1A siRNAs. GAPDH served as protein loading control. (B) Fold change of mRNA transcript levels of ANKS1A in HUVEC after transfection with non-silencing control siRNA or smart pool of ANKS1A siRNAs. This had no effect on proliferation of endothelial cells measured by BrdU incorporation (C) and migration (D). (E) Sprouting of HUVEC spheroids in a collagen matrix was not altered after ANKS1A knockdown. *, p < 0.05; **, p < 0.01, n = 3 experiments. Bar graphs show mean values, error bars indicate SD.

**Fig 4:**
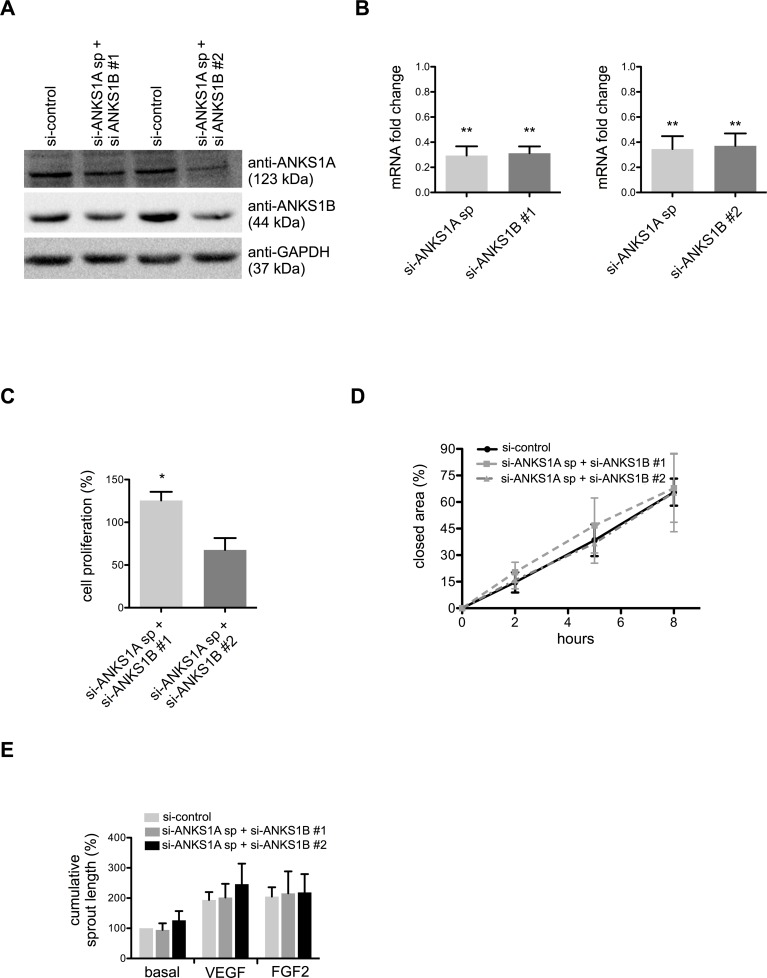
Combined silencing of ANKS1A and ANKS1B does not affect endothelial migration and angiogenesis. (A) Representative Western blot for HUVEC protein lysates probed with anti-ANKS1A and anti-ANKS1B after transfection with non-silencing control siRNA or simultaneous transfection with ANKS1B and a pool of ANKS1A siRNAs. GAPDH served as protein loading control. (B) Quantitative realtime analysis of ANKS1A and ANKS1B mRNA expression in HUVEC treated with the indicated siRNAs. (C) Proliferation and (D) wound closure of HUVEC was not impaired after simultaneous silencing of ANKS1A and ANKS1B. (E) Sprouting angiogenesis under basal conditions, VEGF (25 ng/ml) or FGF2 (25 ng/ml) stimulation in collagen gels was not significantly changed after silencing of ANKS1A and ANKS1B. *, p < 0.05; **, p < 0.01, n = 3 experiments. Bar graphs show mean values, error bars indicate SD.

ANKS1B and ANKS1A contain a similar domain structure (Figure A in S1 File) and we hypothesized that reduction of ANKS1B expression might be compensated by ANKS1A. Therefore, we analysed the effects of silencing ANKS1A alone and in combination with ANKS1B knockdown. ANKS1A expression was successfully silenced in HUVEC with a pool of four different siRNAs (Fig 3A and 3B). Analyses of BrdU incorporation (Fig 3C), wound closure (Fig 3D and Figure A in S2 File) and spheroid-based sprouting angiogenesis (Fig 3E and Figure B-D in S2 File) revealed no consistent and significant differences compared to control. Secondly, HUVEC were transfected with siRNAs against ANKS1A and ANKS1B (Fig 4A and 4B). No or only minor significant changes were observed in endothelial cell proliferation (Fig 4C), migration (Fig 4D and Figure A in S3 File) and sprouting angiogenesis (Fig 4E and Figure B-D in S3 File).

Furthermore, knockdown of ANKS1A and ANKS1B alone as well as in combination had very slight, but no biological relevant influence on the transcription of KRIT1 or the Notch ligand DLL4 and the Notch target gene HEY1 (Figure A-C in S4 File) that are regulated by KRIT1 and ICAP1 in HUVEC [11,13,19]. Taken together, silencing of ANKS1A and ANKS1B expression did not phenocopy the effects of KRIT1 or ICAP1 manipulation in primary endothelial cells [11,13].

### Silencing ANKS1B expression enhances endothelial cell permeability

**Fig 5:**
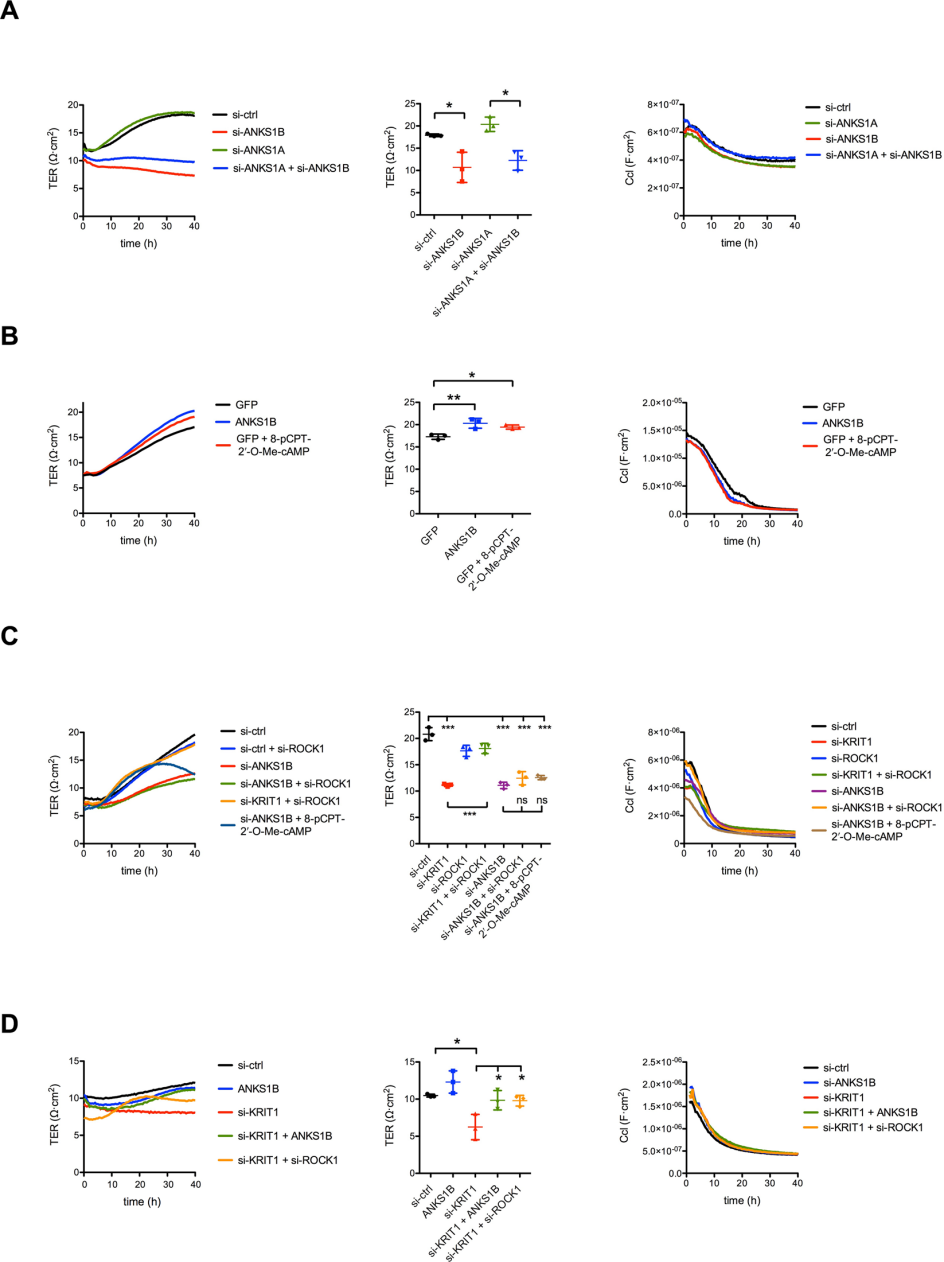
ANKS1B promotes endothelial cell permeability. (A) Representative graphs of TER and Ccl measurement after ANKS1B- and ANKS1A-silencing via siRNA transfection, alone or in combination. Measurements with a second siRNA against ANKS1B showed comparable results. (B) Representative TER/Ccl measurement comparing ANKS1B overexpressing endothelial cells to GFP expressing +/- 5 μM cyclic AMP treated cells. Adenoviral overexpression of ANKS1B leads to a significantly increased electrical resistance in HUVEC comparable to values reached with cyclic AMP treatment. (C) Representative graphs of TER and Ccl measurement after ROCK1-, KRIT1- and ANKS1B-silencing via siRNA transfection, Knockdown of ROCK1 or addition of 8-pCPT-2′-O-Me-cAMP could not rescue the drop of electrical resistance due to ANKS1B silencing. In contrast silencing of ROCK1 lead to an rescue of almost 100% in KRIT1-silenced endothelial monolayers. (D) Representative graph of a transendothelial electrical resistance (TER) and the corresponding capacity (Ccl) measurement. Endothelial cells deficient of KRIT1 were treated with an adenovirus overexpressing ANKS1B. The increase of electrical resistance is comparable with KRIT1-deficient endothelial cells silenced for ROCK1. *, p < 0.05; **, p < 0.01, ***, p < 0.001, n = 3 experiments. Bar graphs show mean values, error bars indicate SD.

Loss of KRIT1 does not only cause excessive angiogenesis but also hyperpermeability in cell culture and mouse models. We analysed endothelial barrier functions in freshly isolated primary endothelial cells (passage 0) upon silencing of ANKS1B. As readout we determined the transendothelial electrical resistance (TER) and corresponding capacity (ccl). This method allows the real time measurement of paracellular permeability and monolayer formation. Compared to control there was a dramatic decrease of transendothelial resistance after ANKS1B knockdown indicating impaired barrier functions (Fig 5A). The ccl values were similar in both groups indicating the formation of a dense monolayer (Fig 5A). Next we overexpressed ANKS1B with adenoviral vectors. This led to approximately 20% increase in TER indicating improved barrier tightness. This was similar as the increase of TER after treatment with the cAMP analog 8-pCPT-2′-O-Me-cAMP (Fig 5B), which activates the guanine nucleotide exchange factor Epac to tighten endothelial cell junctions [30]. As such, ANKS1B was identified a novel protein that helps to maintain endothelial barrier functions.

The loss of KRIT1 leads to impaired function of the GTPase Rap1 and hyperactivation of Rho kinase and this is supposed to be the major cause for hyperpermeability in CCM models [1]. We silenced Rho kinase (ROCK1) expression alone or in combination with ANKS1B (Fig 5C and Figure A in S5 File). The knockdown of ROCK1 could not rescue the drop of electrical resistance due to ANKS1B silencing. This effect was also not reversed by addition of 8-pCPT-2′-O-Me-cAMP after ANKS1B-silencing. In addition, silencing of ANKS1B had no effect on RhoA- and Rac1-activation levels (RhoA: 98.72% ± 46.02, p = 0.9532; Rac1: 114% ± 34.7, p = 0.2901). This indicates that ANKS1B acts in contrast to KRIT1 not via RhoA and RhoA-controlled kinases to limit endothelial permeability.

Lastly we investigated if forced ANKS1B expression could rescue the barrier defects upon silencing of KRIT1. siRNA-mediated knockdown of KRIT1 expression led to a pronounced reduction of transendothelial resistance as expected (Fig 5D). Similar to ROCK1-silencing, could the forced expression of ANKS1B improve permeability upon silencing of KRIT1 (Fig 5D). KRIT1 knockdown did not affect ANKS1B protein expression (Figure A in S5 File). Therefore, binding of ANKS1B to KRIT1 is probably not necessary to improve endothelial cell barrier functions.

In summary the experiments revealed that ANKS1B is a novel binding protein of KRIT1. ANKS1B did not influence angiogenesis but it was integral to maintain endothelial cell barrier functions.

## Discussion

Cerebral cavernomas are among the most frequent vascular malformations in the central nervous system. The majority of patients carry mutations in the *CCM* genes. Several important functions of CCM proteins have been unravelled in recent years, most notably the control of endothelial cell quiescence, endothelial to mesenchymal transistion and permeability [1]. However, it is still unclear how the proteins execute their functions since they do not contain catalytically active domains. This study was aimed at identifying new interacting proteins of KRIT1 with a yeast 2-hybrid screening approach. The vast majority of positive clones encoded for CCM2 and ICAP1, two well-known interacting proteins [15,31–33]. ANKS1B was identified as a novel binding protein. The experiments revealed that this enigmatic protein was dispensable to control angiogenesis but it significantly contributed to enhance endothelial cell barrier functions.

Binding of KRIT1 to other proteins may generate protein complexes that execute the functions of this important protein [1]. KRIT1 contains three NPxY/F motifs, four ankyrin repeats and a C-terminal a band 4.1/ezrin/radixin/moesin (FERM) domain, and all of them are typically used for protein interactions. KRIT1 binds with the first NPxY motif to the PTB domain of ICAP1 while the others mediate binding of CCM2 [15,31–33]. Our study implicates that also the PTB domain of ANKS1B can be bound by KRIT1. This raises questions about how many PTB-containing proteins can be recruited at the same time, how they compete for binding. It is difficult to address these questions with the available techniques. There is still a lack of suitable, commercial antibodies for CCM proteins and we also failed to detect specific expression patterns of ANKS1A and ANKS1B in endothelial cells by using a set of commercially available antibodies.

A striking observation in the study was the selective requirement of ANKS1B for permeability but not angiogenesis control. This is similar to the GTPase Rap1, another binding protein of KRIT1. Rap1 is bound via the FERM domain and it stabilizes endothelial cell junctions. KRIT1 associates with microtubules, [34] and Rap1 binding transfers KRIT1 from microtubules to cell-cell junctions [35]. Rap1 cannot reverse the increased cell permeability upon silencing of KRIT1 [36]. In our study, forced ANKS1B expression could enhance transendothelial resistance also upon silencing of KRIT1 expression indicating that ANKS1B acts independently of KRIT1 and RhoA. Taken together, ANKS1B was identified as a new contributing factor for endothelial barrier functions.

## Supporting Information

S1 File KRIT1 interacts with ANKS1B. (A) Protein domain diagrams of ANKS1A, ANKS1B and KRIT1. (B) HEK293T cells were transiently transfected with plasmids encoding the indicated fusion proteins. After cell lysis protein A-tagged proteins were precipitated and the interaction with a luciferase-tagged protein was measured by bioluminescence. The positive interaction between the two proteins is indicated by a LUMIER intensity ratio value of 3 or above. The strong, known interaction between JUN and FOS was used as a positive control. (n = 3 experiments). (C) Coomassie blue stained SDS-PAGE of GST-NPxY-F1-F3 fusion proteins obtained from E.coli BL21 cells 3 hours after IPTG induction. (D) Western blot of cell lysates and GST pull-down elution fractions probed with anti-ANKS1B and anti-GST. ***, p < 0.001. Bar graphs show mean values, error bars indicate SD.
(TIFF)

S2 File ANKS1A does not alter the proliferation and migration behaviour of endothelial cells. (A) Representative images showing wound closure of HUVEC after ANKS1A-silencing. (B) Microscopical images of HUVEC spheroids after ANKS1-silencing. (C) The individual sprout length and (D) sprout number under basal conditions, VEGF (25 ng/ml) or FGF2 (25 ng/ml) showed no significant difference. N = 3 experiments. Scale bar equals 100 μm. Bar graphs show mean values, error bars indicate SD.
(TIFF)

S3 File Combined silencing of ANKS1A and ANKS1B does not strongly effect migration and proliferation of endothelial cells. (A) Representative images of wound-closure after silencing ANKS1A and ANKS1B in primary endothelial cells. (B) Microscopical images of HUVEC spheroids after simultaneous knockdown of ANKS1A and ANKS1B under basal or VEGF- and FGF2-inducing conditions. (C) Quantification of the individual sprout length and (D) sprout number of HUVEC spheroids as shown in (B). N = 3 experiments. Scale bar equals 100 μm. Bar graphs show mean values, error bars indicate SD.
(TIFF)

S4 File Knockdown of ANKS1A and ANKS1B has no biological relevant influence on the transcription of KRIT1 or the Notch ligand DLL4 and the Notch target gene HEY1. Quantification of mRNA transcript levels of DLL4, HEY1 and KRIT1 in HUVEC after silencing (A) ANKS1B, (B) ANKS1A or (C) ANKS1B and ANKS1A in combination. *, p < 0.05, n = 3 experiments. Bar graphs show mean values, error bars indicate SD.
(TIFF)

S5 File (A) Representative Western blot for HUVEC protein lysates probed with anti-ROCK1 after transfection with non-silencing control siRNA or ROCK1-siRNA. GAPDH served as protein loading control. (B) Representative Western blot of HUVEC protein lysates probed with anti-KRIT1 and anti-ANKS1B after KRIT1-silencing by siRNA transfection. GAPDH served as protein-loading control.
(TIFF)

S1 Table List of primer sequences used for quantitative realtime PCR analysis in this study. (TIFF)

S2 Table Putative binding partners of KRIT1 detected in a yeast two-hybrid screening assay (TIFF)

## Abbreviations

ANKS1B: ankyrin repeat and sterile alpha motif domain-containing protein 1B
CCM: Cerebral cavernous malformation
ECBM-2: endothelial basal medium
ECGM-2: endothelial growth medium
HUVEC: human umbilical vein endothelial cell
KRIT: Krev interaction trapped protein
LUMIER: luminescence-based mammalian interactome mapping
PTB: phosphotyrosine binding domain
SAM: sterile alpha motif domain
