# Supplementary figures and images for: ANKS1B Interacts with the Cerebral Cavernous Malformation Protein-1 and Controls Endothelial Permeability but Not Sprouting Angiogenesis
Source: PLoS One. 2015 Dec 23;10(12):e0145304. doi: 10.1371/journal.pone.0145304 (PMC4699217; doi:10.1371/journal.pone.0145304)

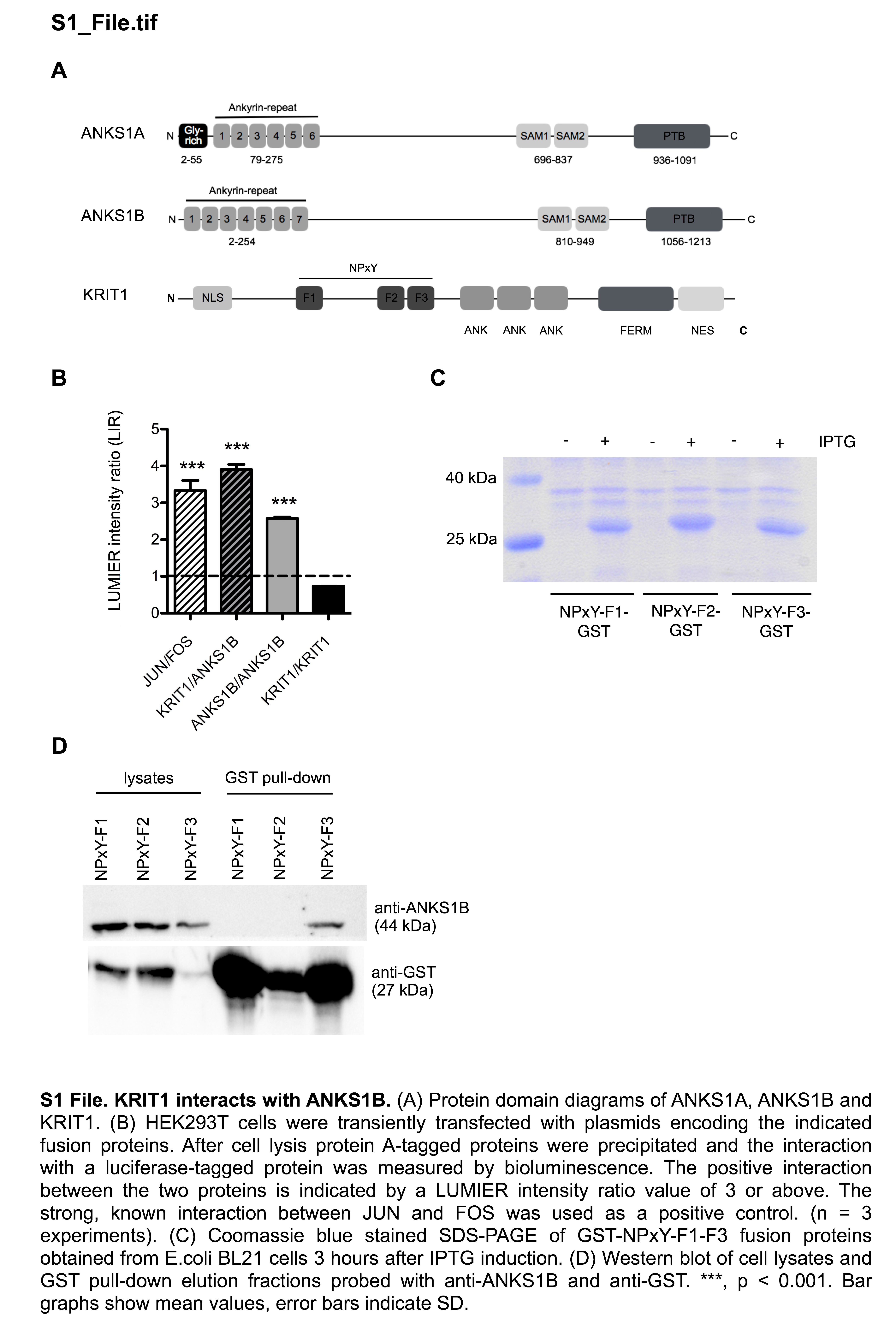

Supplement: S1 File — (A) Protein domain diagrams of ANKS1A, ANKS1B and KRIT1. (B) HEK293T cells were transiently transfected with plasmids encoding the indicated fusion proteins. After cell lysis protein A-tagged proteins were precipitated and the interaction with a luciferase-tagged protein was measured by bioluminescence. The positive interaction between the two proteins is indicated by a LUMIER intensity ratio value of 3 or above. The strong, known interaction between JUN and FOS was used as a positive control. (n = 3 experiments). (C) Coomassie blue stained SDS-PAGE of GST-NPxY-F1-F3 fusion proteins obtained from E.coli BL21 cells 3 hours after IPTG induction. (D) Western blot of cell lysates and GST pull-down elution fractions probed with anti-ANKS1B and anti-GST. ***, p < 0.001. Bar graphs show mean values, error bars indicate SD. (TIFF) [file pone.0145304.s001.tiff]

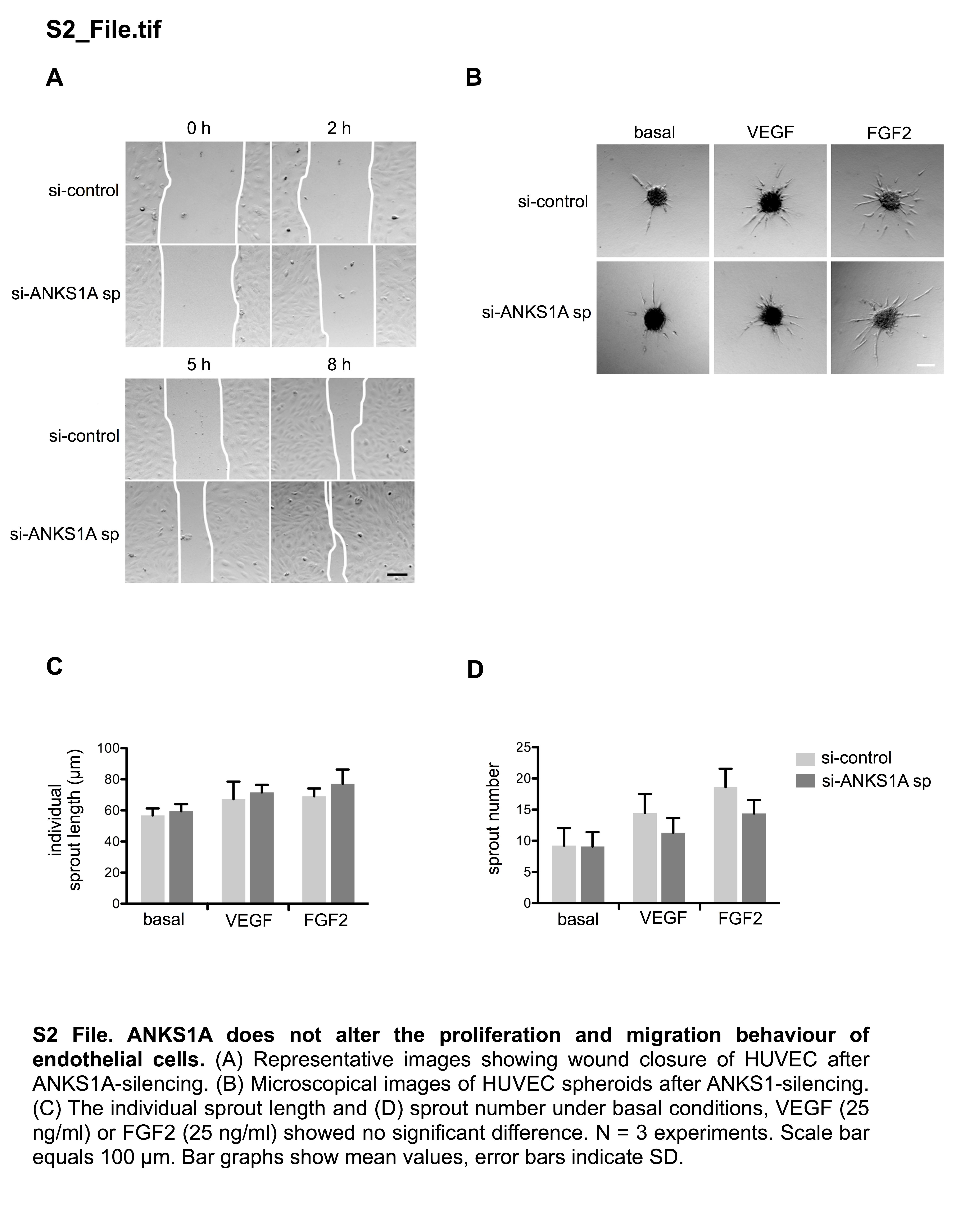

Supplement: S2 File — (A) Representative images showing wound closure of HUVEC after ANKS1A-silencing. (B) Microscopical images of HUVEC spheroids after ANKS1-silencing. (C) The individual sprout length and (D) sprout number under basal conditions, VEGF (25 ng/ml) or FGF2 (25 ng/ml) showed no significant difference. N = 3 experiments. Scale bar equals 100 μm. Bar graphs show mean values, error bars indicate SD. (TIFF) [file pone.0145304.s002.tiff]

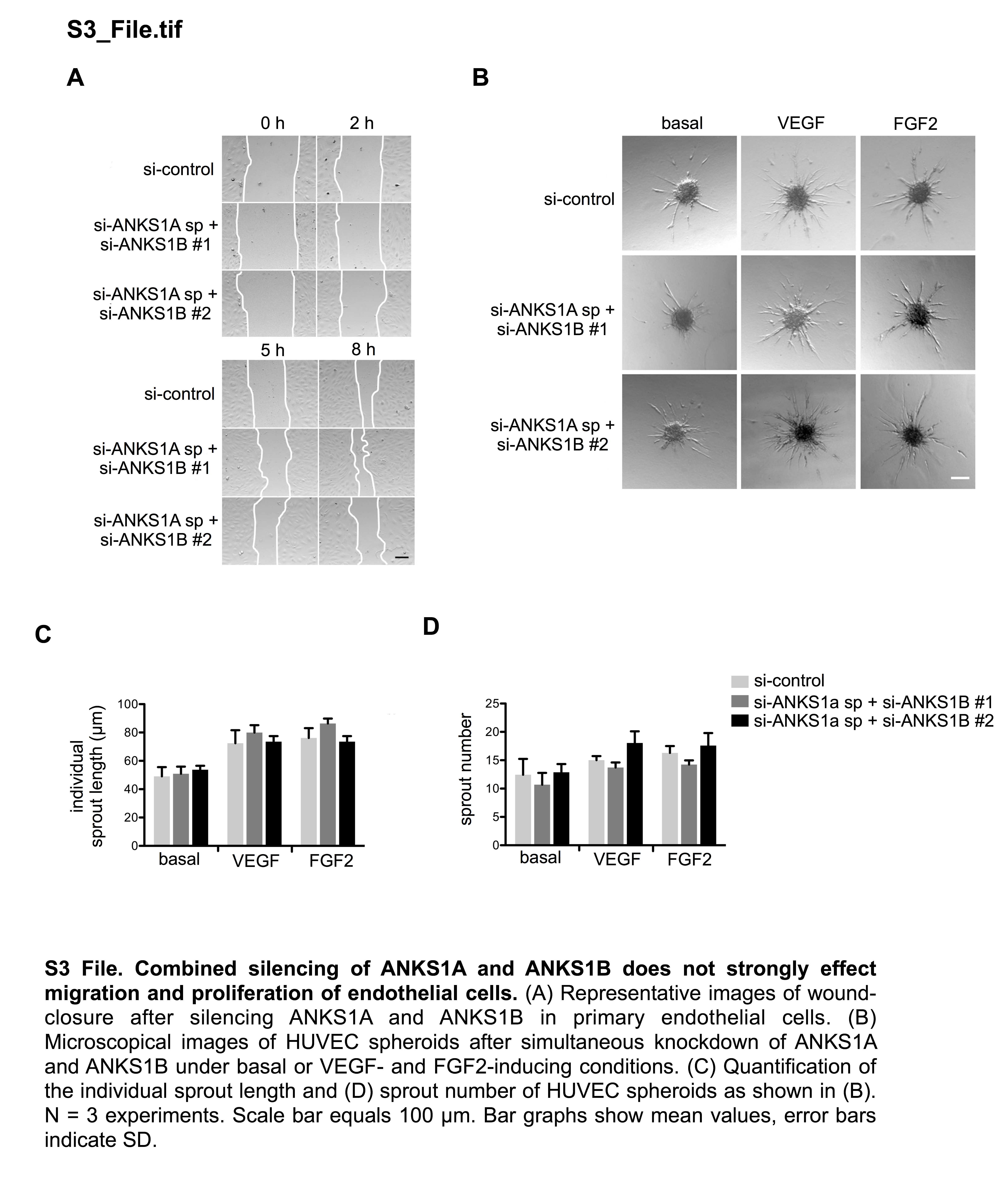

Supplement: S3 File — (A) Representative images of wound-closure after silencing ANKS1A and ANKS1B in primary endothelial cells. (B) Microscopical images of HUVEC spheroids after simultaneous knockdown of ANKS1A and ANKS1B under basal or VEGF- and FGF2-inducing conditions. (C) Quantification of the individual sprout length and (D) sprout number of HUVEC spheroids as shown in (B). N = 3 experiments. Scale bar equals 100 μm. Bar graphs show mean values, error bars indicate SD. (TIFF) [file pone.0145304.s003.tiff]

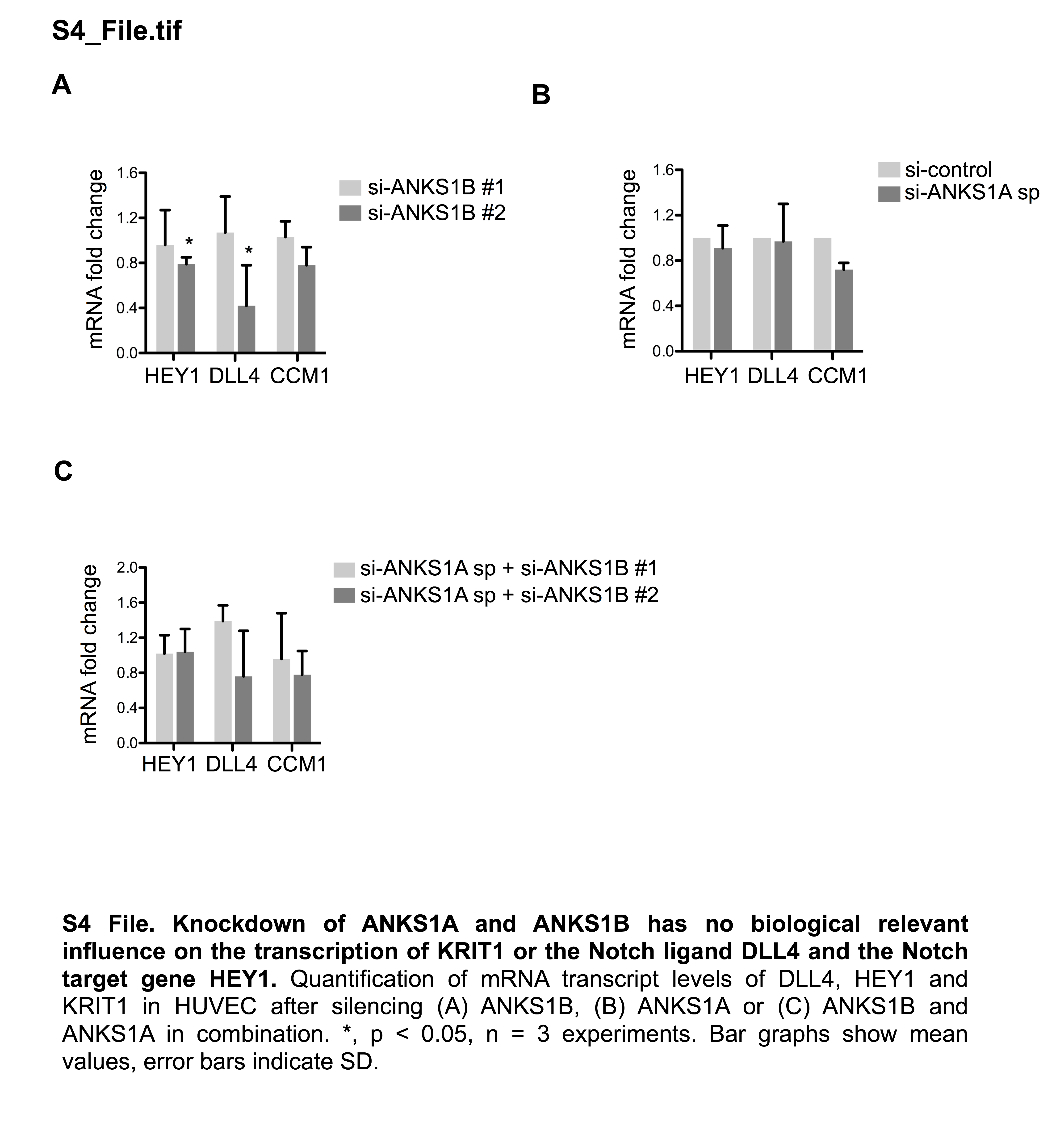

Supplement: S4 File — Quantification of mRNA transcript levels of DLL4, HEY1 and KRIT1 in HUVEC after silencing (A) ANKS1B, (B) ANKS1A or (C) ANKS1B and ANKS1A in combination. *, p < 0.05, n = 3 experiments. Bar graphs show mean values, error bars indicate SD. (TIFF) [file pone.0145304.s004.tiff]

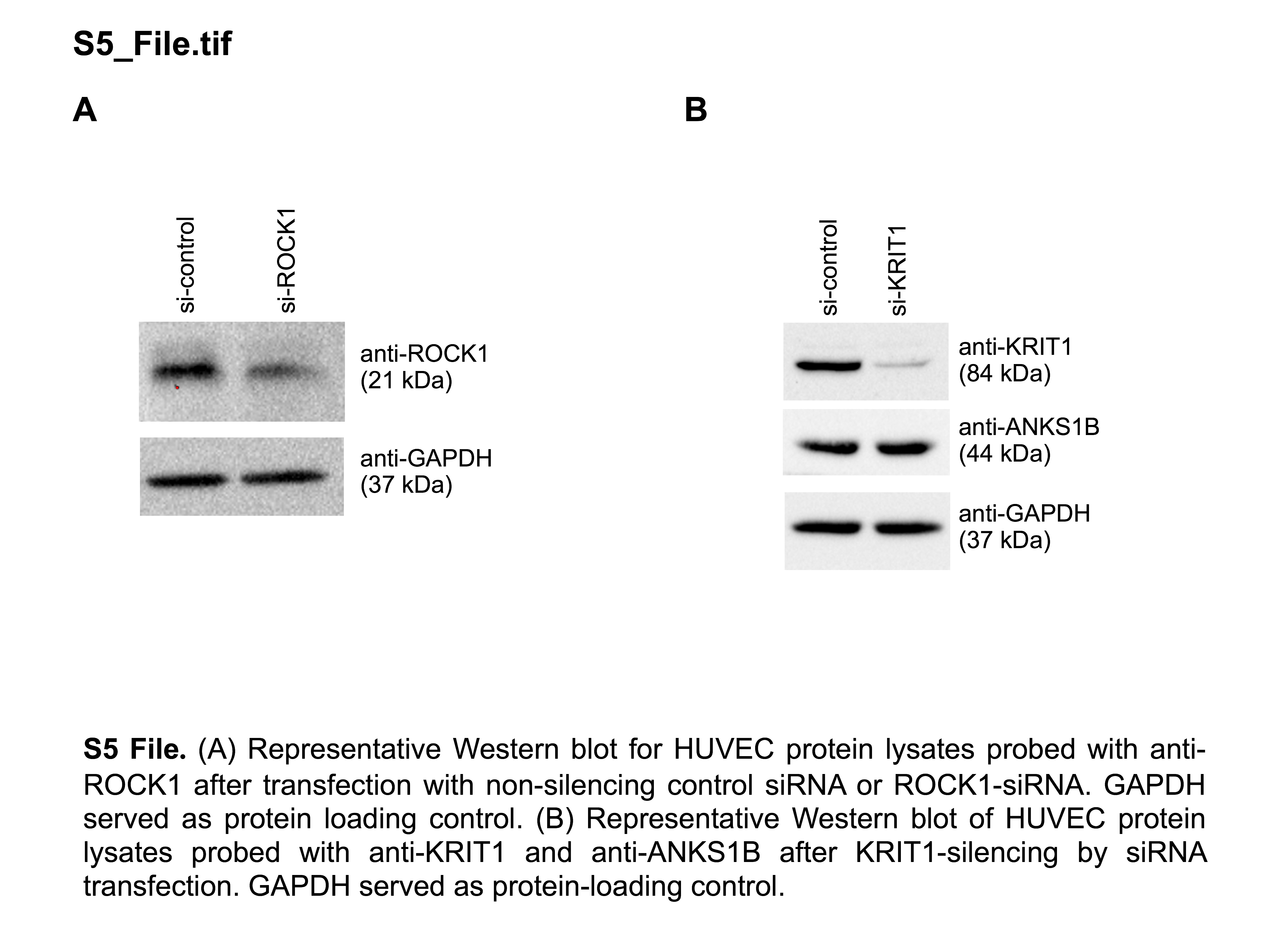

Supplement: S5 File — (A) Representative Western blot for HUVEC protein lysates probed with anti-ROCK1 after transfection with non-silencing control siRNA or ROCK1-siRNA. GAPDH served as protein loading control. (B) Representative Western blot of HUVEC protein lysates probed with anti-KRIT1 and anti-ANKS1B after KRIT1-silencing by siRNA transfection. GAPDH served as protein-loading control. (TIFF) [file pone.0145304.s005.tiff]

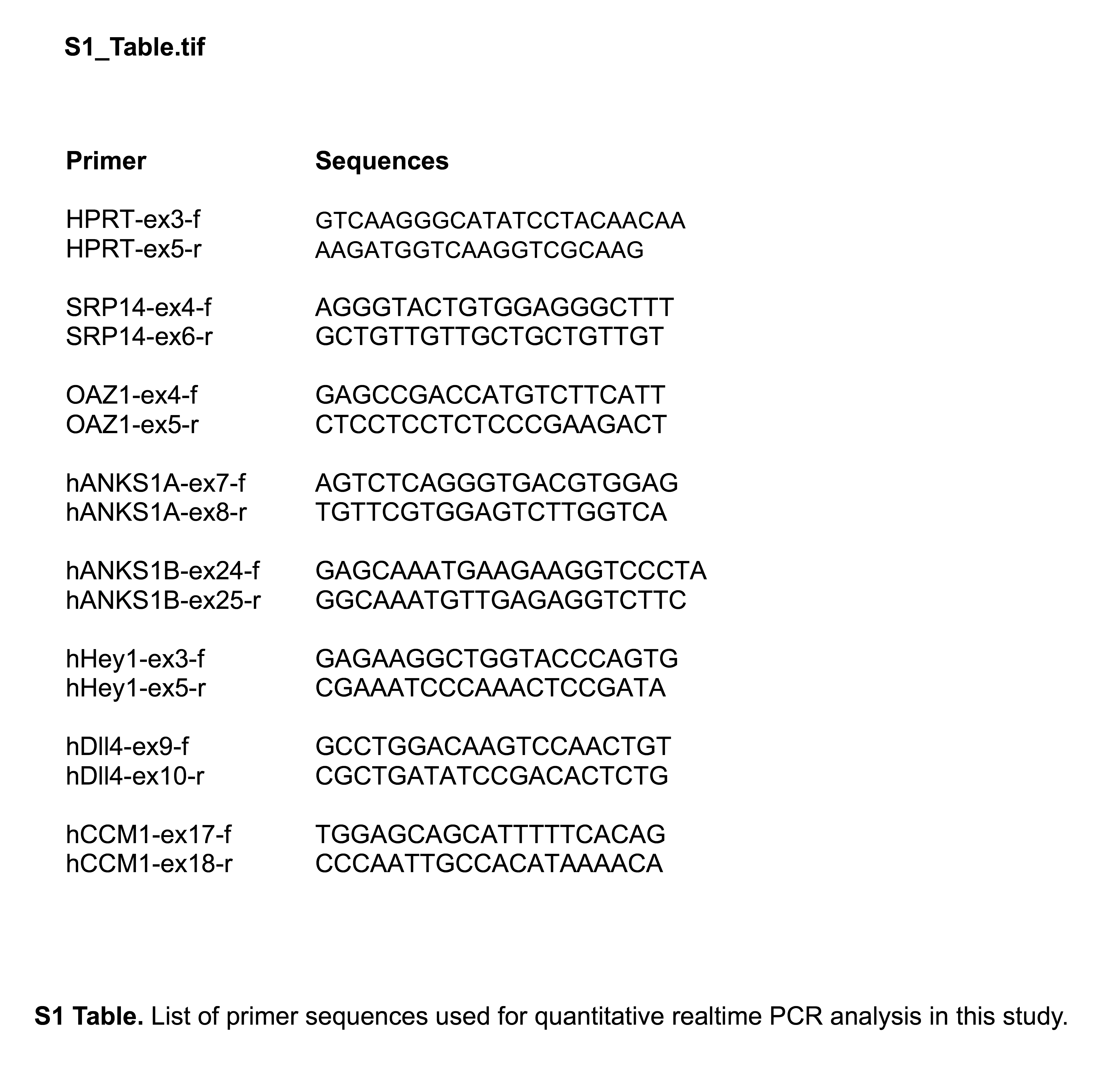

Supplement: S1 Table — (TIFF) [file pone.0145304.s006.tiff]

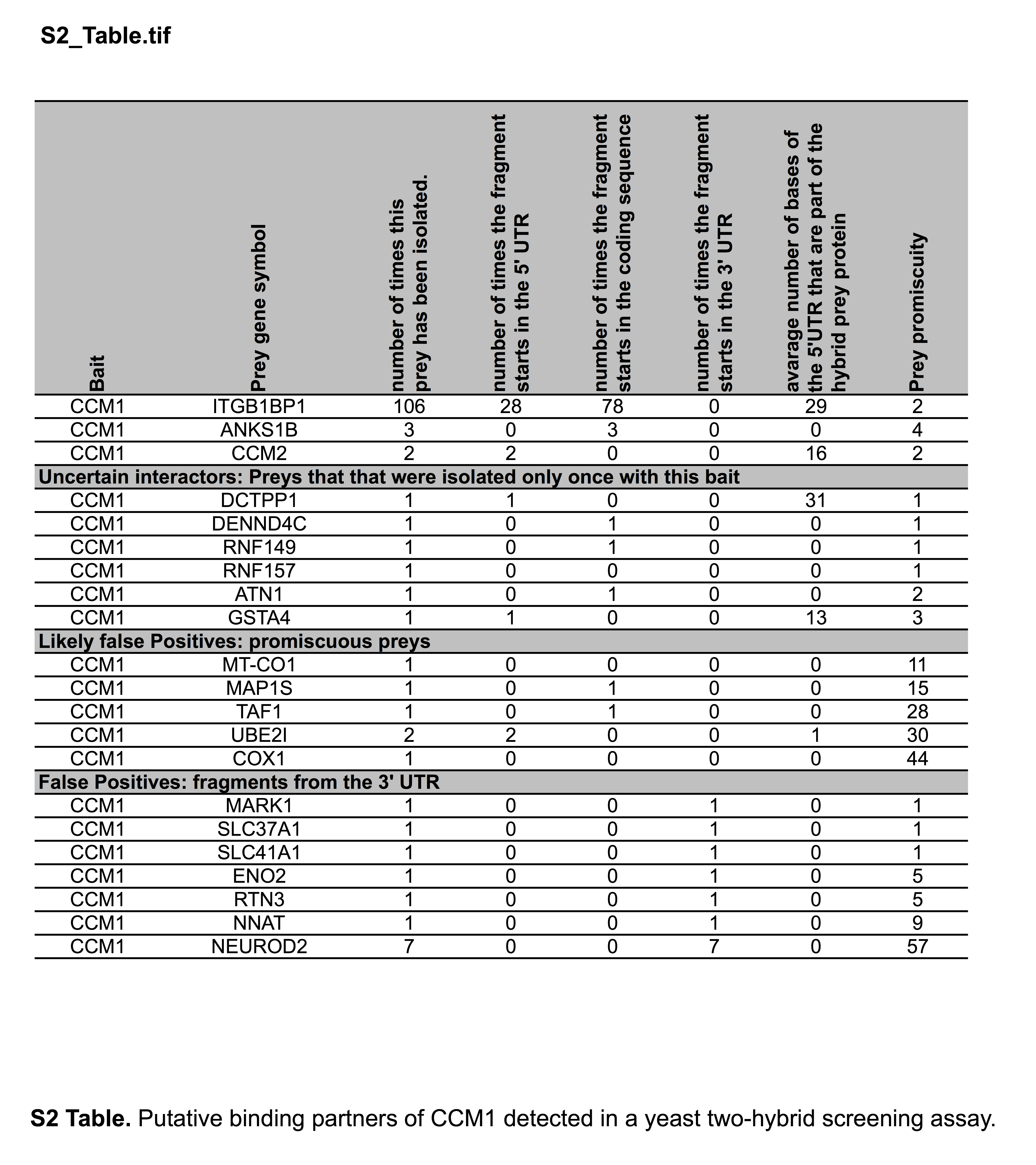

Supplement: S2 Table — (TIFF) [file pone.0145304.s007.tiff]
